# Color Transfer Algorithm between Images Based on a Two-Stage Convolutional Neural Network

**DOI:** 10.3390/s22207779

**Published:** 2022-10-13

**Authors:** Min Xu, Youdong Ding

**Affiliations:** Shanghai Film Academy, Shanghai University, Shanghai 200072, China

**Keywords:** VGG19, CNN, reference image, color transfer, emotional color

## Abstract

A color transfer algorithm between images based on two-stage convolutional neural network (CNN) is proposed. The first stage network is based on VGG19 architecture as the backbone. The reference image-based color transfer (RICT) model was used to extract the features of the reference image and the target image, so as to realize the color transfer between them. The second stage is based on progressive convolutional neural network (PCNN) as its backbone. The palette-based emotional color enhancement (PECE) model is adopted to enhance the emotional coloring of the resulting image by comparing the palette, emotional value and the proportion of each color of the reference image. Through five sets of experiments, it is proved that the visual effect processed by our model is obviously better than several main colorization methods in both subjective evaluation and objective data. It can be applied to various complex scenes, and in the near future, it can also be better applied to the fields of digital restoration of old image archives, medical image coloring, art restoration, remote sensing image enhancement, infrared image enhancement and other fields.

## 1. Introduction

Color transfer is one of the key research topics in the field of digital image processing, and its related technologies can be applied in image coloring and color reproduction, the digital restoration of old image archives, medical image coloring, art restoration, remote sensing image enhancement, infrared image enhancement and other fields. In 2016, Google, Facebook, Adobe, Tencent and many other internet companies had already announced that they would start research projects related to image stylization. Color transfer technique is based on the reference image $R^{\prime }$ and the target image *T* to synthesize a new image $T^{\prime }$, and it requires $T^{\prime }$ to retain the content of *T* and inherit the color of $R^{\prime }$. Therefore, content preservation and color transfer based on semantic information need to be solved before the algorithm is designed. For content preservation, we need to find a way to change the color of the image well without causing any geometric change in the image. Reinhard [1] addressed this challenge with global color transformation, but it could only handle simple color transfers because it could not model the effects of spatial changes. For color transfer, we should respect the semantic of the scene. In [2], convolutional neural network (CNN) and Markov random field(MRF) were adopted for regional matching, but other unrelated regions of the style image were ignored, resulting in a great difference between the generated image and the expected style. It can be seen that color transfer between images is still a research difficulty for the near future. It is of great practical significance to continue to optimize existing color transfer algorithms or to explore new methods. Gatys et al. [3] have verified that VGG19-based style transfer can also perform color transfer, which also inspires the idea of our work in this paper.

Color enhancement of the image or video can be divided into two categories: fully automatic color enhancement [4,5,6] and semi-automatic color enhancement of user interaction. Among them, there are four ways of user interaction: image coloring based on color hints [7,8], image coloring based on reference images [1,9,10,11,12,13,14,15,16,17,18,19,20,21,22], image coloring based on palette [23,24] and image coloring based on text [25,26]. The image coloring based on the reference image requires the user to provide a reference image as input, and then find the feature matching between the reference color image and the target gray image with the help of the neural network model, and complete the copy and transfer of the corresponding color.

As early as 2001, Reinhard et al. [1] proposed a color transfer algorithm between color images by using the orthogonality of matrices. Later, color transfer has attracted extensive attention from scholars at home and abroad. Many scholars have improved or proposed new color transfer algorithms on the basis of the original algorithm. Inspired by these color transfer algorithms, Welsh et al. [9] achieved color transfer of grayscale images by matching pixel brightness and texture information of target images and reference images. However, to find the best matching point, we need to traverse all the pixels of the color image, which is quite time-consuming. In order to shorten the time and achieve more accurate local color transfer, Chang et al. [10] proposed to first classify images according to the color, and then match the pixels of target images to the pixels of reference images in the same category. Gupta et al. [11,12,13] proposed that users need to provide or use an algorithm to retrieve a reference image that is semantically similar to the target image from the internet, and then use feature matching to identify the corresponding relationship between pixels in the two images to shorten the time. Since the effectiveness of features is often significantly affected by local features of the image, these traditional methods are more likely to combine multiple low-level features to improve the matching performance. The fact is that low-level features cannot capture the semantic information of the image, so they have a poor color enhancement effect on complex texture images.

Recent studies have used deep learning to learn the semantic information of images, and establish the mapping relationship of pixels to achieve color enhancement. Li et al. [14] realized the automatic coloring of grayscale images by using dictionary matching and sparse reconstruction, all of which are performed at the superpixel level. Vondrick et al. [15] proposed a self-supervised model that learns to track targets by learning video coloring tasks. Li et al. [16] proposed an image coloring method that automatically divides the image area into uniform and non-uniform areas, and selects the appropriate feature vector for each block of the target image to determine the color. Finally, the results are merged together through MRF to improve the consistency. He et al. [17] proposed the first example-based local coloring model. This is achieved by first looking for a reference image in the database similar to the target image, and then using an end-to-end CNN to achieve image coloring. Xiao et al. [18] designed the network as a pyramid structure to facilitate the transfer of color distribution from the low layer to the high layer, and took into account semantic information and details in the coloring. The model no longer produces a fixed color image. Fang et al. [19] proposed that multi-level features of each superpixel should be extracted from the two images, and the most appropriate color of each target should be determined by using variational method. Li et al. [20] proposed an automatic coloring method based on local texture matching. Its innovation is that it introduces a new idea of cross-scale matching, upper and lower color position distribution, and a color propagation method based on confidence weighting to make the edge coloring better.

**Figure 1:**
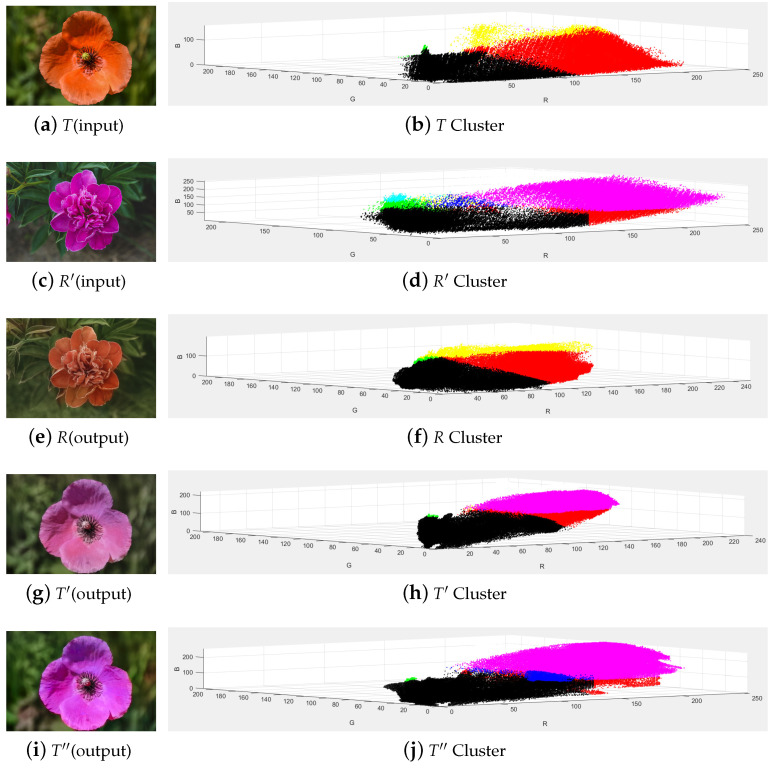
Color tranfer effect of ours.

Color transfer between images means to transfer the color of reference image to the target image through a mapping, so as to transform the color of the target image from one distribution to another distribution, and requires the minimum cost of transformation, which is essentially the optimal transmission problem. Our target is to transform grayscale images or images with little color into color images with high contrast, clear details, clear colors, and better visual and sensory effects, so as to improve the ability of human eyes to distinguish image details. In this paper, a color transfer algorithm between images based on two-stage CNN is proposed, and the resulting images are shown in Figure 1. The image $T^{\prime }$ output by the neural network in the first stage has the content of *T* and the attributes of $R^{\prime }$, with better effect. The image $T^{\prime \prime }$ output by the neural network in the second stage contains more emotional factors, which can express content beyond the image and meet the needs of users better.

The contributions of this paper include: (1) A new deep neural networks (DNN) model that can generate multi-target coloring results is proposed. The model can generate different color palettes according to different reference images, and then produce different coloring effects. (2) Color transfer is only applied to the color space, which can suppress the image distortion and generate satisfactory coloring effects in various scenes. (3) Regardless of whether the reference image is related to the target image, the model can deliver reasonable colors and prevent spillover effects, such as the texture of the building not being transferred to the sky.

## 2. Methods

**Figure 2:**
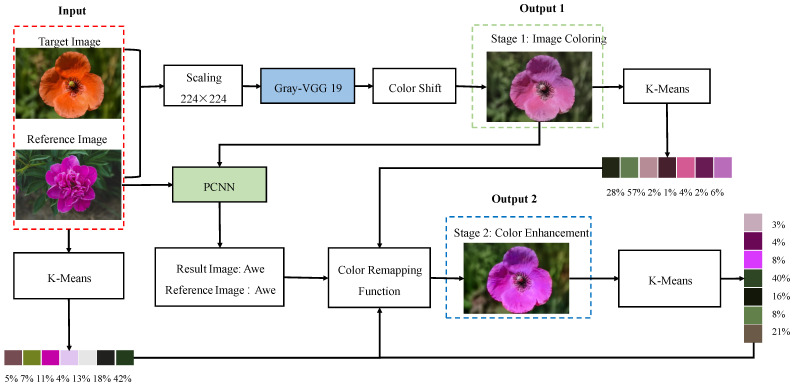
Block diagram of our model.

The architecture of the entire neural network is composed of two stages: the reference image-based color transfer (RICT) model and the palette-based emotional color enhancement (PECE) model, as shown in Figure 2. The network model in the first stage can independently color the target image. The second stage of the network model is to adjust the emotional color of the resulting image, that is, the user can modify the emotional color of the image by editing the palette. The second stage is the emotional enhancement of the coloring result of the first stage, which can make the colored image into the emotional result that the user wants to express.

### 2.1. Reference Image-Based Color Transfer

Colorists are classified as having a technical post because it is not easy to design a set of natural and harmonious color combinations and apply them properly to an image. The purpose of the RICT model is to extract the attribute features of the reference image and apply it to the target image with semantically related content. Before constructing the model, the following two problems need to be solved: First, calculate the semantic correlation between the two images. This paper uses the gray-VGG19 [27] to extract the detailed features of the images and perform feature matching. Second, transfer colors based on similarity rules. The RICT network architecture is shown in Figure 2, the input is the target image $T_{L}\in^{R^{H\times W\times 1}}$ and the reference image $R_{Lab}^{\prime }\in R^{H\times W\times 3}$. Next, the network uses the pre-trained VGG19 to extract the depth feature maps of the two images, and calculates the semantic similarity between the two to obtain the two-way mapping function $\Phi_{T↔R}$. Finally, this information is used to propagate the correct colors to color blocks or pixels with the same semantics.

#### 2.1.1. Feature Extraction and Feature Matching

As *T* and $R^{\prime }$ have different visual effects, it is difficult for the network to directly learn the mapping from *T* to $R^{\prime }$. However, it can be decomposed into two steps: (1) $T\to T^{\prime }$ is a mapping of the same position; (2) $T^{\prime }\to R^{\prime }$ is a color mapping. Since $T\to T^{\prime }$, $R\to R^{\prime }$ is an alignment mapping of the same position points, $\Phi_{t\to r}$ is defined as *T* or $T^{\prime }$ to *R* or $R^{\prime }$. Similarly, $\Phi_{r\to t}$ is a mapping from *R* or $R^{\prime }$ prime to *T* or $T^{\prime }$ prime. Assuming that the point *p* of the graph *T* is mapped to the point $p^{\prime }$ of the graph $R^{\prime }$, then: $T\left(p\right)=R\left(\Phi_{t\to r}\left(p\right)\right),T^{\prime }\left(p\right)=R^{\prime }\left(\Phi_{t\to r}\left(p\right)\right)$. At the same time, *p* and $p^{\prime }$ should be same, namely: $p^{\prime }=\Phi_{t\to r}\left(p\right)$. To strengthen the symmetry constraint, bidirectional constraint can be added: $\Phi_{r\to t}\left(\Phi_{t\to r}\left(p\right)\right)=p,\Phi_{t\to r}\left(\Phi_{r\to t}\left(p^{\prime }\right)\right)=p^{\prime }$.

The output of VGG−19 network is a pyramid with 5 layers (i=1,2…,5) of feature maps, with feature maps $F_{T}^{i}$ and $F_{R^{\prime }}^{i}$ for each layer. Assuming the highest level $F_{T}^{5}=F_{T^{\prime }}^{5},F_{R^{\prime }}^{5}=F_{R}^{5}$, the first step is to calculate the mapping of the fifth layer by using nearest-neighbor Field Search: $\Phi_{t\to r}^{5}$ and $\Phi_{r\to t}^{5}$. The second step is to modify $F_{R^{\prime }}^{5}$ according to $\Phi_{t\to r}\left(5\right)$ to obtain $F_{R^{\prime }}^{5}\left(\Phi_{t\to r}\left(5\right)\right)$. As layer pooling is adopted between layers of VGG network, the $F_{R^{\prime }}^{5}\left(\Phi_{t\to r}\left(5\right)\right)$ obtained is half of the size of $F_{T}^{4}$. Then, it is calculated layer by layer until we obtained the $\Phi_{t\to r}^{1}$, $\Phi_{r\to t}^{1}$, $F_{T}^{i}$ and $F_{R^{\prime }}^{i}$ of the first layer.

For the four feature maps $\left(F_{T}^{i},F_{R}^{i},F_{T^{\prime }}^{i},F_{R^{\prime }}^{i}\right)$ of layer *i*, it is defined as follows:

(1) $$\Phi_{t\to r}^{i}\left(p\right)=argmin_{q}\sum_{x\in N\left(p\right),y\in N\left(q\right)}\left({∥{\bar{F}}_{T}^{i}\left(x\right)-{\bar{F}}_{R}^{i}\left(y\right)∥}^{2}+{∥{\bar{F}}_{T^{\prime }}^{i}\left(x\right)-{\bar{F}}_{R^{\prime }}^{i}\left(y\right)∥}^{2}\right)$$

Among them, ${\bar{F}}^{i}\left(x\right)=\frac{F^{i}\left(x\right)}{\left|F^{i}\left(x\right)\right|}$, N(p) is a small patch centered on point *p*. When i=5,4,3, patch takes 3×3; when i=2,1, patch takes 5×5.

When we obtain $\Phi_{t\to r}^{i}$, we need to construct the i−1th layer $\left(F_{T}^{i-1}\right)$:

(2) $$F_{T^{\prime }}^{i-1}=F_{T}^{i-1}\cdot W_{T}^{i-1}+H_{R^{\prime }}^{i-1}\cdot \left(1-W_{T}^{i-1}\right)$$

Among them, $H_{R^{\prime }}^{i-1}$ is obtained by $F_{R^{\prime }}^{i}$ through transformation. That is, we use the *i*th layer to obtain $\Phi_{t\to r}^{i}$, and obtain $\Delta_{t\to r}^{i}$ after upsampling, and then act on $F_{R^{\prime }}^{i}$ to get $F_{R^{\prime }}^{i}\left(\Delta_{t\to r}^{i}\right)$ as $H_{R^{\prime }}^{i-1}$. Convolution, pooling, and other operations have been performed between the i−1th layer and the *i*th layer, and $H_{R^{\prime }}^{i-1}$ and $F_{T}^{i-1}$ are not aligned. To obtain $F_{T}^{i-1}$, you need to calculate $F_{T}^{i}$ and reconstruct $F_{R^{\prime }}^{i}$ in advance. If $CNN_{i-1}^{i}\left(\cdot \right)$ is defined as a sub-network between layer i−1 and layer *i*, then $CNN_{i-1}^{i}\left(H_{R^{\prime }}^{i-1}\right)$ obviously has to be as close to $F_{R^{\prime }}^{i}\left(\Delta_{t\to r}^{i}\right)$ as possible. Therefore, we can obtain: $\varepsilon ={∥CNN_{i-1}^{i}\left(H_{R^{\prime }}^{i-1}\right)-∥}^{2}$, $F_{R^{\prime }}^{i}\left(\Delta_{t\to r}^{i}\right)$ can be approximated.

#### 2.1.2. Color Transfer

Obviously, the larger the $W_{T}^{i-1}$, the more we want $F_{T}^{i-1}$ to use more content structure of $F_{T}^{i}$ and less detailed features of $F_{R^{\prime }}^{i}$. Now, we define:

(3) $$W_{T}^{i-1}=\alpha^{i-1}\cdot M_{T}^{i-1}$$

Where $M_{T}^{i-1}$ is obtained by the sigmoid function after normalization of $F_{T}^{i-1}$. That is:

(4) $$M_{T}^{i-1}\left(x\right)=\frac{1}{1+exp\left(-\kappa \times \left({\left|F_{T}^{i-1}\left(x\right)\right|}^{2}-\tau \right)\right)},\kappa =300,\tau =0.05$$

$\Phi_{t\to r}^{i-1}$ must be obtained by fine adjustment of the features of 4 images in the i−1 layer through nearest-neighbor field search. In the nearest-neighbor field search of the i−1th layer, a random search is performed only within a certain range around the point *p* of the mapping relationship $\Delta_{t\to r}^{i-1}$, so as to fine-tune $\Delta_{t\to r}^{i-1}$ to obtain $\Phi_{t\to r}^{i-1}$. For the layer 4,3,2,1, the search range radius is 6,6,4,4, respectively. Calculations are performed layer by layer until we obtain $\Phi_{t\to r}^{1}$, and then use $\Phi_{t\to r}^{1}$ as $\Phi_{t\to r}$. This is because there is no pooling between $\Phi_{t\to r}^{1}$ and the input layer, and the spatial size is the same. When $\Phi_{t\to r}$ is obtained, the colored image $T^{\prime }$ can be obtained:

(5) $$T^{\prime }\left(p\right)=\frac{1}{n}\sum_{x\epsilon N\left(p\right)}\left(R^{\prime }\left(\Phi_{t\to r}\left(x\right)\right)\right)$$

where n=5.

For the backlight image *I*, the target image is first enhanced through the color estimation model (CEM) [28]:

(6) $$I^{GE}=\frac{1-f_{CEM}\left(I\right)}{1+f_{CEM}\left(I\right)}$$

where $f_{CEM}\left(I\right)=e^{-\frac{\lambda_{I}}{M_{I}}}$, $\lambda_{I}$ is the adjustment parameter ($\lambda_{I}=1.33$), and $M_{I}$ is the gray mean of the image I. By using the CEM to enhance the backlight image globally, the overall brightness of the input image can be improved and the color and detail information of the image can be restored.

### 2.2. Palette-Based Emotional Color Enhancement

Images can not only affect people on an emotional level, but also directly express people’s emotions. An emotion can be expressed in multiple color combinations, and the same color combination can also express different emotions. However, when the proportion of colors in the image is similar, the emotions displayed in the image will be similar. The purpose of the PECE model is to modify the color palette of the resulting image to enhance its emotional color based on the various data of the reference image. Before constructing the model, the following three problems need to be solved: First, the emotional value of the resulting image and the reference image is calculated. CNN extracts color features, texture features, and content features for image emotion classification, so as to train and learn the image emotion and simulate to solve the problem of subjective evaluation of image emotion. Second, we obtain the color palette of the resulting image and the reference image, and count the proportion of each color in the respective screen. K-Means algorithm is used to re-cluster the foreground palette and the background palette into “5 + 2” color palettes. Third, color remapping is performed. In these tasks, the palette is expressed in the form of a color wheel, which can display specific colors, color names, and proportions, and it has good artistic reference value.

#### 2.2.1. Emotional Computation of Images

**Figure 3:**
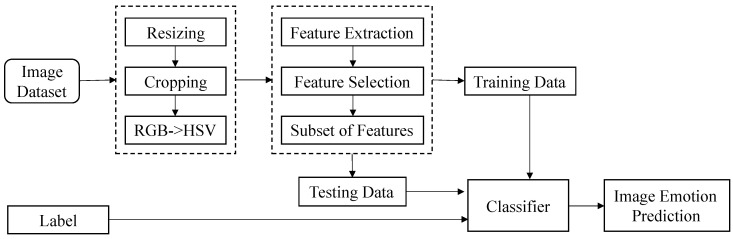
Flow chart of image sentiment classification. The PCNN uses the feedback mechanism to filter out the incorrectly labeled data in the training set, which further improves the ability of image sentiment classification. These eight types of emotions are amusement, anger, awe, contentment, disgust, excitement, fear, sadness.

You et al. [29] proposed a progressive convolutional neural network (PCNN) model based on CNN, which uses CNN to continuously learn the semantic features of the image itself to classify the image emotions positively and negatively. This paper will directly use the PCNN to train and realize the classification of eight kinds of emotions in images on ArtPhoto dataset [30] and FlickrEmotion dataset [31]. The main process is shown in Figure 3, including image preprocessing, feature extraction and feature selection, classifier design and learning.

(1) Image Preprocessing

Segmentation, enhancement, and morphological processing of the input original image can remove redundant information in the input image, filter the noise, and enhance the information features in the image. The size of each image after preprocessing is adjusted to 224×224, which makes the classifier better extract the features of the input image.

(2) Feature Extraction and Feature Selection

Machajdik et al. [30] proposed that from the perspective of psychology and art theory, color, texture, and composition can be used as features to express image emotions. This article will use the color histogram based on HSV blocks [32] to extract the color features of the image, that is, first convert the RGB value of each pixel to HSV. Then, the three HSV values are weighted and summed to obtain a value to represent the color feature. Local binary pattern (LBP) features [33] are used to describe the local texture features of the image. Histogram of oriented gradients (HOG) features [34] are used to represent the appearance and shape of local objects in the image. Haar-like features (content features) [35] are used to recognize human faces. These features are combined to obtain a 47-dimensional feature. There are many methods of feature selection, such as principal component analysis (PCA) [36], exhaustion [37], heuristic search [38], or random search [39].

(3) Loss Function

The loss function is the key to affecting the image emotion classifier and model learning effect. For the loss function in CNN, the nonlinear error function is:

(7) $$E\left(k,\beta ,W,b\right)=\frac{1}{2}\sum_{n=1}^{l}{∥h_{n}-y_{n}∥}^{2}$$

where *k*, β, *W*, and *b* in turn represent the convolution kernel parameters in the convolutional layer that are continuously optimized and updated during the back propagation (BP) in CNN, the weight coefficient of the down-sampling layer, the weight of the fully connected layer, and the bias value. $h^{{}_{n}}$ represents the output value of the network layer, $y^{{}_{n}}$ represents the corresponding expected output value. According to the final output loss function, the entire network is fine-tuned in the reverse direction, and the BP algorithm is used to adjust the parameter values in each layer, so that the loss reaches the minimum value, and the final classification is closer to the expected value.

#### 2.2.2. Palette Generation

**Figure 4:**
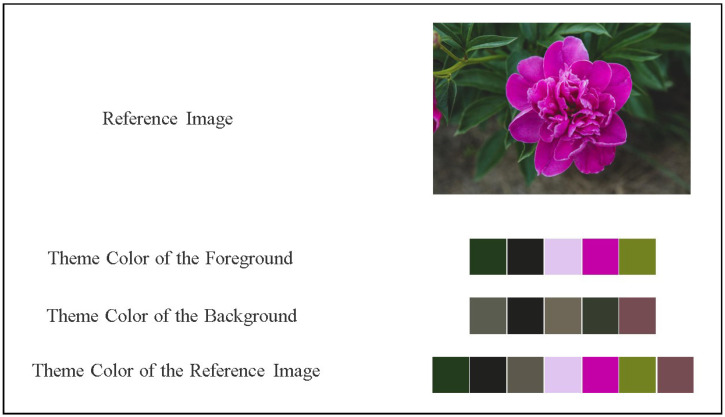
“5 + 2” color palette.

Considering that the color distribution is related to the object, GrabCut [40] can be used to extract the foreground and background of the image, and then K-Means [41] is used to re-cluster the foreground and background palettes into “K + 2” (default K=5) color palette. The effect is shown in Figure 4:

(1) GrabCut

This involves selecting a quadrilateral box and using the color image in the box as an input parameter of GrabCut, indicating that the pixels in the box may belong to the foreground, but the part outside the box must belong to the background. This article will use the mask image as an input parameter of GrabCut to mark the foreground and background of the image.

(2) K-Means

K-Means is used to cluster the foreground and background of the image, and then integrate the palettes of the two by 5:2 to identify the theme color of the image. The step of K-Means is to determine *K* objects as the initial clustering center by histogram peak filtering method, then calculate the distance between each object and each seed clustering center, and assign each object to the clustering center that is closest to it. The cluster centers and the objects assigned to them represent a cluster. For each sample assigned, the cluster center of the cluster is recalculated according to the existing objects in the cluster. This process is repeated until a termination condition is met. The termination condition can be that no objects are reassigned to different clusters, no cluster centers change again, and the sum of squared errors is locally minimum.

(3) Determine the Range of Color Blocks

The RGB color space of the image is converted to the HSV color space, and then the corresponding range of the color is set to be extracted by comparing with the reference table of HSV to get the number of pixels of various colors and determine the range of color blocks (for example, h is red within 156–180).

(4) Color Ratio Calculation

Divide the number of pixels of a certain color in the image with the total number of pixels, and the obtained proportion can be roughly determined as the color ratio of that color in the image.

#### 2.2.3. Palette-Driven Based on Color Remapping

Palette-driven based on color transfer mainly modifies the brightness of pixels with the help of the brightness transfer function $f_{L}$, modifies the corresponding color value with the help of the color transfer function $f_{ab}$, and finally combines the color emotion with the image coloring to produce an image with emotional tone. As for the mapping function *f*, it is necessary to satisfy the interpolation properties, range properties, function continuity, one-to-one relationship, and the monotonicity of brightness [23].

(1) Brightness Transfer Function

A weighted combination of the two most recent palette entries is used. The first operation is to extract the brightness $L_{i}^{\prime }$ corresponding to the modified palette, and perform a monotonic sorting operation on the corresponding colors $C_{i}$ in each palette. Define the original brightness *L* color value of the seven-color palettes of the input image to reach $L_{i<j}<L_{j}$, and the brightness value of the seven-color palettes after color editing to meet $L_{i<j}^{\prime }<L_{j}^{\prime }$. The palette $C_{i}^{\prime }$ can be edited by the user at will, but one condition must always be maintained: $L_{i<j}^{\prime }=max\left(L_{j}^{\prime },L_{j-1}^{\prime }\right)$.

(2) Color Transfer Function

Single palette: Assume that only one color *C* is gathered in the palette. For any color *x*, there is $x^{\prime }=f_{1}\left(x\right)$, that is, the user can modify the color *C* to $C^{\prime }$ through the color transfer function $f_{1}$. For the function $f_{1}$, it needs to meet the one-to-one color transfer rule, which can be divided into two steps: The first step is to find $f_{b}$, that is, the intersection of ray and color gamut boundary from *C* to $C^{\prime }$ direction; the second step is to determine whether $x_{0}=x+C^{\prime }-C$ is in the color gamut. If the range of $x_{0}$ is in the color gamut, it is far from the color gamut boundary, and $x_{b}$ is the position where the parallel rays from *x* to $x^{\prime }$ intersect the color gamut boundary. If $x_{0}$ is beyond the color gamut boundary, but it is very close to the color gamut boundary, then $x_{b}$ can be defined as the point from $C^{\prime }$ to $x_{0}$ where it intersects the gamut boundary. Finally, let $f_{1}\left(x\right)=x^{\prime }$, the point from *x* to $x_{b}$ is expressed as:

(8) $$\frac{∥x^{\prime }-x∥}{∥C^{\prime }-C∥}=min\left(1,\frac{∥x_{b}-x∥}{∥C_{b}-C∥}\right)$$

When it is far from the boundary of the gamut, $f_{1}\left(x\right)$ is proportional to $C^{\prime }-C$ and the maximum ratio is 1. In this case, the color of the palette is changed in parallel. As it is very close to the boundary, the offset problem is $C^{\prime }$, which helps to achieve the desired palette change in these areas.

*K* color Palette: When *K* colors are accumulated in the palette, $f_{1}\left(x\right)$ should be promoted to include K(K>1) theme colors. The strategy adopted is to define K transfer functions, each of which is equivalent to the function $f_{1}\left(x\right)$, and then mix them and weigh them by proximity. The weight is:

(9) $$f\left(x\right)=\sum_{i}^{k}W_{i}\left(x\right)f_{i}\left(x\right)$$

Among them, $\sum_{i}^{k}W_{i}\left(x\right)=1$. Finally, the weights are obtained by using the least squares method. As follows:

(10) $$W_{i}\left(x\right)=\frac{1}{{\left|C_{j}-x\right|}^{2\alpha }}$$

where the scalar parameters meet α≤1, and $C_{j}$ are the color values of the palette. If you want to change the colors of five palettes at the same time, compatibility and coordination between the five colors is very important, and the color-enhanced image cannot appear with color penetration or color inconsistency. Therefore, after the color conversion equation is determined, all pixels in the input image are updated next: $I_{\left(x,y\right)}^{\prime }=I_{\left(x,y\right)}+C^{\prime }-C$, $I_{\left(x,y\right)}$ is the pixel of the input images, $I_{\left(x,y\right)}^{\prime }$ is the pixel after the color update, $C^{\prime }$ is the five-color color combination in the selected database, and *C* is the color in the image palette.

### 2.3. Objective Function and Network Training

The objective function needs to be determined by two necessary conditions: First, the color of the reference image with the highest similarity is applied first; second, even if there is no reliable reference image, the network can learn the inherent color association between the two based on the gray information. This paper directly uses the loss function in the literature [21] to train the network. It is a multi-task network including a chromaticity branch and a perceptual branch, but both branches use the same network structure *C* and weight θ.

When training the chroma branch, we input $T_{L}$ and $T_{ab}^{\prime }$ into the network to generate the result $P_{ab}^{T}$: $P_{ab}^{T}=C\left(T_{L},sim_{T↔R},T_{ab}^{\prime };\theta \right)$. $P_{ab}^{T}$ is colored based on $T_{ab}^{\prime }$, and the chromaticity information $T_{ab}$ should be restored when the correct sample and color are selected. The chromaticity error adopts smooth $L_{1}$: $L_{chrom}\left(P_{ab}^{T}\right)=\sum_{p}L_{1}\left(P_{ab}^{T}\left(p\right),T_{ab}\left(p\right)\right)$. Using $L_{1}$ can avoid obtaining a mean solution in the fuzzy coloring problem.

When training the perceptual branch, we input $T_{L}$ and $R_{ab}^{\prime }$ into the network to predict chromaticity information $P_{ab}$: $P_{ab}=C\left(T_{L},sim_{T↔R},R_{ab}^{\prime };\theta \right)$. Perception error: $L_{perc}\left(P_{ab}\right)=\sum_{p}{∥F_{p}\left(p\right)-F_{T}\left(p\right)∥}^{2}$. $F_{p}\left(p\right)$ is the feature map obtained by $P_{Lab}$ through the VGG relu5_1 layer, and $F_{T}$ is the feature map obtained by $T_{Lab}$ through the VGG relu5_1 layer. Perceptual error can eliminate semantic differences caused by incorrect coloring, and it can enhance the robustness of two different reasonable colors. The parameters can be optimized in the following ways:

(11) $$\theta^{\prime }=argmin_{\theta }L_{chrom}\left(P_{ab}^{T}\right)+\alpha L_{perc}\left(P_{ab}\right)$$

Among them, the parameter α is used to indicate the relative weight between the two branches, and it is set to 0.005.

## 3. Results

### 3.1. Experimental Environment

The network structure is built on the well-known tensorflow. Tensorflow converts the network into a calculation graph, and realizes learning and training through BP. Both the hardware and software configuration of our computer is shown in Table 1.

### 3.2. Dataset

The dataset for training the color transfer model is ImageNet [42]. In order to make the network robust to any reference image, the similarity values of the reference image and the resulting image sampled in this paper are distributed during 0−1. In the training phase, we randomly exchange resulting images and reference images to enhance the data.

There are two datasets for training sentiment classification networks. The first dataset is ArtPhoto, which is a public dataset for image sentiment classification downloaded from art websites. The second is FlickrEmotion, which is downloaded from the Flickr website and annotated to form a complete image dataset. Table 2 is the specific classification number of these two datasets:

### 3.3. Color Quality Evaluation

According to the visual perception theory and the purpose of color transfer, the color transfer algorithm between images should be divided into two aspects for evaluation: First, the result image should obtain the color characteristics of the reference image well; Second, the result image should keep the content of the input image unchanged. “content” refers to the shape and structure information of the image, such as the boundary of the region in the image. Therefore, quantitative evaluation indexes in this paper includes: peak signal-to-noise ratio (PSNR) [43], structure similarity (SSIM), quaternion structural similarity (QSSIM) between the result image and the target image [44], colorfulness similarity (CS) between the result image and the reference image [45]. The color brightness transfer function evaluation [46] is discussed, which is meaningful for the use of color transfer in Lab color space. Qualitative evaluation is reflected in the fact that we invited 20 college students with normal vision (10 women and 10 men), ranging in age from 20 to 30 years old, the task is to let them compare our method with other methods.

### 3.4. Experimental Results and Analysis

We compare our results with those seven of previous methods, including Iizuka [4], Larsson [5], Zhang [6], Reinhard [1], Welsh [9], Xiao [18], and Lee [47]. The first three are fully automatic methods and the last four are example-based methods. Since our network has no constraints on input images and reference images, we prepare many sets of real color images as our reference images. First, we compare our method with state-of-the-art colorization methods (Section 3.4.1). Then, we make a comparative analysis of the influence of different color combinations on the final coloring results of the model, and finally explain the rationality of the seven-color combination in this paper (Section 3.4.2). Next, we also conduct a set of studies on the impact of emotion classification models on image effect, and point out that the PCNN has an immeasurable contribution to this paper (Section 3.4.3). Next, we use various reference images, including randomly generated color palettes, to evaluate the robustness of the proposed model (Section 3.4.4). Finally, we directly compare the running time of several models to process an image (Section 3.4.5).

**Figure 5:**
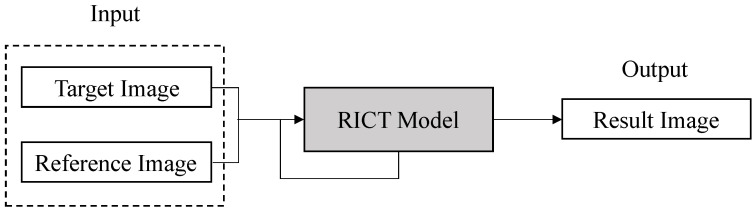
Flow char of experiment.

The experimental process is shown in Figure 5, the target image and the reference image of the same scene are used to train the entire network model, and the resulting image is obtained. The two indicators of computational amount/FLOPS (namely, the number of operations of the model, time complexity) and memory access/Bytes (namely, the number of parameters of the model, space complexity) are often used to judge the performance of deep learning models. The number of parameters of our model is 195 M, and the number of operations of our model is 20.3 M. Due to the large number of parameters, we set it to save every sixty epochs, and finally only save the model with the least loss and the highest accuracy on the validation set. It takes around 1.8 s for coloring an image (512×512) in average.

#### 3.4.1. Comparison with Different Methods

**Figure 6:**
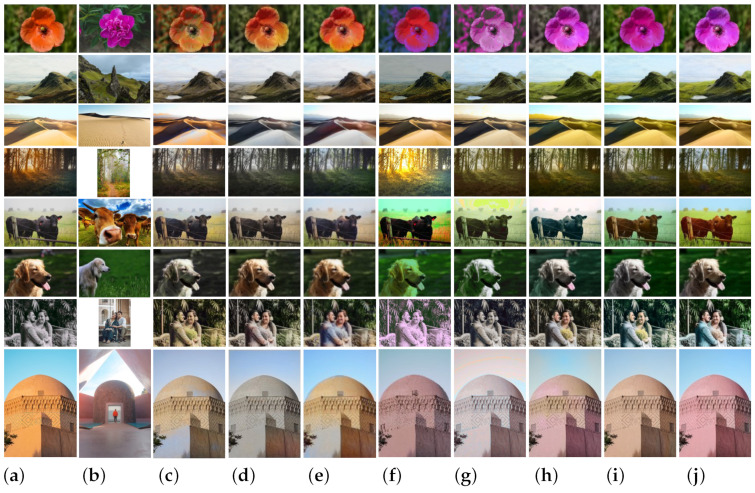
Comparison of coloring effects of different algorithms. The first column is the target image (TI), and the second column is the reference image(RI). (**a**) TI. (**b**) RI. (**c**) [4]. (**d**) [5]. (**e**) [6]. (**f**) [1]. (**g**) [9]. (**h**) [18]. (**i**) [47]. (**j**) Ours.

Figure 6 shows the comparison of coloring effects of eight algorithms on eight groups of images. Given a target image Figure 6a and a reference image Figure 6b, different algorithms can be used to generate an output image Figure 6f–j that has the same scene as the target image and a similar style to the reference image. Among them, Figure 6c–e are the coloring results automatically generated by the model according to the grayscale information of the target image itself. The resulting image has nothing to do with the given reference image, but is closer to the original color of the target image. On the whole, these eight different algorithms are very successful in coloring these eight images. If analyzed in detail, we will find the differences between these algorithms. It can be seen from Figure 6c–e that the three fully automatic coloring methods are more accurate and natural for the coloring of simple objects (such as the sky, flowers, trees, grass, etc.), while the coloring of complex objects (such as beach, people, buildings, etc.) is inaccurate or uneven, and there is a problem of color overflow in the processing of different object boundaries (such as water and mountains, sky and lawn, etc.).

As can be seen from Figure 6f, the overall color of the new image generated by Reinhard [1] has significantly changed compared with the original image, but it also produces distortion. Especially when the color of the image is complex, it is difficult for the user to accurately select the sample block, and the algorithm will lose its effect (for example, the coloring of the seventh group of human images). Welsh [9] mainly uses brightness and standard deviation to make the best match, which requires a high consistency between brightness and color correspondence. The mean and variance used by its formula are for the whole image, so it has a very poor effect on the dispersion characteristics of light in the natural environment. As can be seen from Figure 6g, the images colored by the Welsh [9] generally show a single color level, which is not suitable for coloring scenes with complex contents (such as the coloring of the seventh group of human images and the eighth group of buildings). Part of Xiao [18]’s code is improved based on Zhang [6], so his model has achieved good results. This paper proposes a new dense encoding pyramid network, which can predict the color of gray image by analyzing the color distribution of reference image, and the coloring result is very flexible. However, color leakage and color bleaching often occur in areas with open boundaries (such as the coloring of the last three groups of images in Figure 6h. Lee [47] proposes a DNN that leverages color histogram analogy for color transfer. Using semantic segmentation information, this method can process many image pairs with similar semantic content in different scenes, but it may also decrease the correct rate of coloring due to segmentation errors (for example, in Figure 6i, the lake water and the sky are the same color, and the cattle fence and the prairie are the same color). In contrast, our result (Figure 6j) transfers the color of the reference image better, retaining the realism of the output image.

In order to better demonstrate the advantages of this algorithm, the PSNR values, SSIM values, and QSSIM values of the resulting images obtained by each algorithm are compared. By comparing the values in Table 3, it is found that the objective evaluation values of the first three fully automated coloring methods based on the grayscale information of the image itself to predict color information are generally stronger than the latter five example-based color transfer algorithms, but the proposed model is the method with the best objective evaluation effect among the last five algorithms. Although the PSNR value of the resulting image obtained by using the model in this paper is not the best value among eight comparison algorithms, it can be found that most of it is within the top three best values, and is basically not ranked in the bottom three. By comparing the values in Table 4, it is found that the SSIM value of the resulting image obtained by using the model in this paper accounts for five out of the eight best values, and the proportion reaches 62.5%. Even though the other two values are not optimal, they are all within the top three optimal values. By comparing the values in Table 5, it is found that except for the Reinhard [1] and the Welsh [9], the QSSIM values obtained by the other five algorithms are generally good and very similar. Based on the observation of the experimental results in Figure 6 and the comparison of the numerical results listed in Table 3, Table 4 and Table 5, it can be seen that the algorithm proposed in this section has obtained better subjective effects and higher PSNR and SSIM values.

#### 3.4.2. Contrast of Enhancement Effect of Three-Color/Five-Color/Seven-Color/Multi-Color Combination

**Figure 7:**
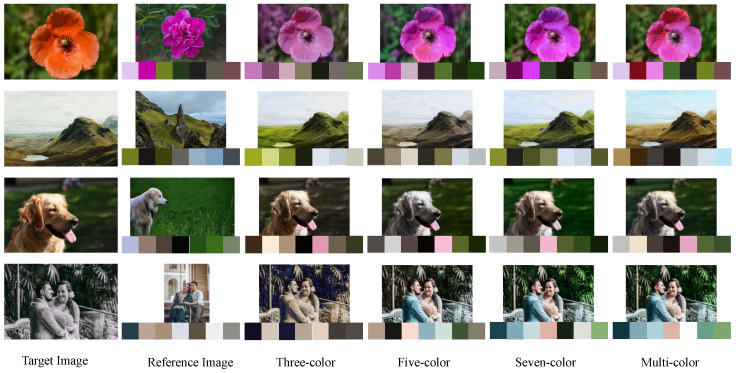
The effect of different color combinations on the coloring effect.

Figure 7 shows the comparison of the coloring results of our model on the following four images when the example-based palette is set to three-color, five-color, seven-color, and multi-color in sequence. According to the final results generated by the model, too few colors in the palette will easily lead to the problem of single color in the new image, while too many colors in the palette will increase the difficulty of calculation. The above problems can be basically solved only when the number of colors is set to seven. Selecting a palette of three-color palettes produces new images that generally exhibit a single color. For example, the background of the first flower is painted the same purple as the flowers, the lake among the mountains is painted the same green as the mountains, the dog’s hair and the lawn are basically the same color, the two people and the trees behind them seem to be only added warm color, so it is hard to see other colors in the image. Selecting a palette of five-color palettes produces new images, and the results are good, but inevitably, there is less color. For example, the background of flowers is dark green, the color of mountains is brown, and the color of dog’s hair is gray, the same color rarely appearing between the transition color is monotonous, there is less color. Choosing a palette of seven-color palettes produces a new image that looks more natural and comfortable than the previous two, and its palette is also closer to that of the reference image. The new images generated by selecting the palette of multi-color combinations also produced better results than the previous two, and were even similar to the results generated by the model selecting the seven-color combination. However, considering that it is difficult to calculate the total number of colors in an image and to find an optimal value to represent the color palette of the image, and choosing a palette with multiple color combinations will increase the computational difficulty of the model. Therefore, this paper sets the palette of the reference image to seven. Experiments show that the results generated by the model with a seven-color palette are no worse than those generated by the model with a multi-color palette, both in terms of effect and color combination.

**Figure 8:**
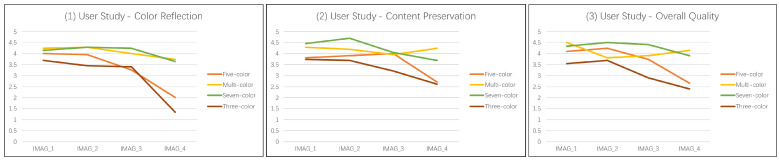
The first user study. Each participant is asked to answer three aspects: color reflection, content preservation, and overall quality.

By comparing the image effects in Figure 7, it is found that the seven-color palette is used in this paper to change the color of the image as a whole, and its color palette is basically similar to that of the reference image. The final effect also meets the needs of the human eye. In order to further verify the above conclusion, 20 people were invited to give subjective scores on the color reflection, content preservation and overall quality of the above four groups of images. By analyzing the data in Figure 8, it can be seen that the result obtained by using the seven-color and multi-color palette is generally better than the result obtained by using the three-color and five-color palette in three indicators, and the resulting images obtained by using seven-color combination palette have the highest score.

In addition to the advantage in subjective visual quality, we also give an objective evaluation based on colorful (C). Table 6 lists the colorful for each reference image and each result image. Since the colorful is not related to the spatial information of the image, that is, an image is scrambled and reorganized, and its colorful remains unchanged. In color transfer, the color visual perception of the result image should be consistent with the color visual perception of the reference image, that is, the smaller the value of CS(res,ref), the better the color transfer result. The data in Table 6 show that the values *C* of the resulting images generated from the three-color and five-color palettes are slightly smaller, and the values *C* of the resulting images generated from the seven-color and multi-color palettes are larger and very similar. Among the four images, the colorful of the resulting image generated by using the seven-color palette has a value as high as 83.764. And one of the other three values, 33.222 is within 33–45, which belongs to the C value that looks more comfortable visually, the other two values are not within this range, they are better than the comparison method in the paper. Based on the analysis of the last column of data in Table 6, the color similarity CS(res,ref)<12 between the result image and the reference image generated by the seven-color palette. This indicates that the two images have similar colors, that is, the color transfer effect of our model is very good.

#### 3.4.3. Influence of Emotion Classification Model on Image Effect

**Figure 9:**
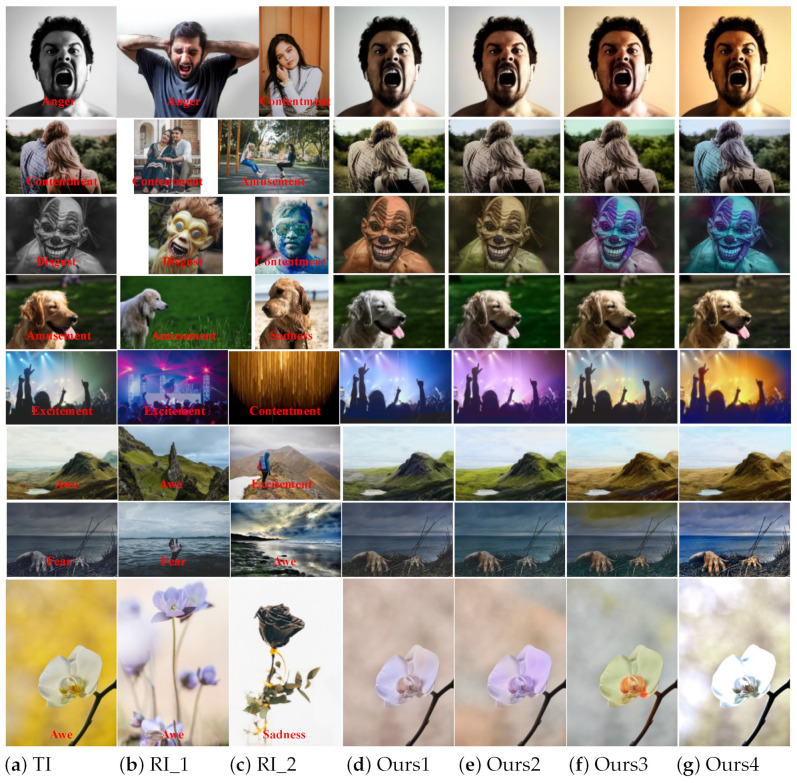
Influence of emotion classification labels on coloring effect. These columns correspond in turn to the target image (TI), reference image 1(RI_1), reference image 2 (RI_2), result 1 (Ours1), result 2 (Ours2), result 3 (Ours3), and result 4 (Ours4). Results 1 and 2 are generated by our model based on reference image 1, results 3 and 4 are generated by our model based on reference image 2. Results 1 and 3 are the generation effects of our model without emotional labels, results 2 and 4 are the generation effects of our model with emotional labels.

In the face of different colors, people will have different psychological reactions such as cold and warm, light and shade, light and heavy, strength, distance, expansion, speed, and so on. This natural reflection of color is a common feature of our people, and is not related to our life experience, age, and climate. It is a more natural reflection of color. We call this natural human response to color the emotional effect of color. Labeling the emotion of an image is inherently subjective and difficult. Different people may experience different emotions from the same image, and the same person may also experience different emotions for the same image at different times. Different images may evoke emotions for different reasons, for example, some images are due to the objects in them, some are the lines in them, and some are some of the compositions. However, it is worth confirming that when the image contains the expressions of the characters, it is difficult for people to change the emotion of the image by changing the color of the image. By observing the eight images in Figure 9, it is found that the emotion of the resulting image is consistent with the original emotion of the target image after the emotion label is added to the first four images, but the final tone is more likely to make people empathize. After adding the emotion label to the last four images, the emotion of the resulting image is no longer completely consistent with the original image, but has changed a lot.

For example, the second image “dog” in Figure 9 also uses brown-based color conversion. Having only the result after adding the emotion label not only ensures that the foreground “dog hair color” is consistent with the reference image, but it also allows the unity of the background color with the reference image, and ensures that the overall look is more natural and the palette is similar to the reference image. For example, the fifth image “concert” and the sixth image “mountains” in Figure 9 showed stronger colors after adding emotional labels, and the resulting images showed inconsistent emotions. As another example, in the last two images in Figure 9, whether to add the emotion label or not has little effect on the color of the resulting image, but its emotion has obviously changed a lot. In the seventh image, when the reference image is labeled with fear, the emotion of the corresponding resulting image is also fear. The resulting image is no longer as fearful as the emotion of the original image, but a little more warm. In the eighth image, when the reference image is a black rose, the emotion of the resulting image generated after labeling the emotion is significantly closer to the emotional color of the reference image. In general, the color of the target image combined with the emotional label of the reference image has little influence on the overall color of the resulting image, but it can maintain the color consistency with the reference image at the critical moment.

**Figure 10:**
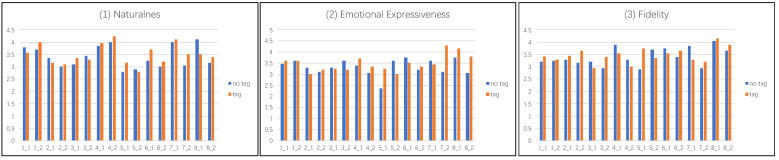
The second user study. Each participant was asked to answer questions in three indicators: naturalness, emotional expressiveness, and fidelity.

To further verify the superiority of our algorithm, we also invited 20 people to score the eight groups of images in Figure 9 according to three indicators: naturalness, emotional expressiveness, and fidelity. As shown in Figure 10, for images with explicit emotions such as the first four images, adding emotion labels or not has little effect on the results. For the fifth and sixth images whose emotions are not easily discernable, the addition of emotion labels still has some effect on the results, so the scores on the three indicators are also unstable, but the overall scores on the three indicators are still slightly higher than those of the model without emotion labels. For the latter two images which feature easy-to-distinguish emotions, adding emotion labels has a great impact on the results, so the scores on the three indicators are generally high. In general, the advantages of adding emotional labels to the model’s color transfer process outweigh the disadvantages.

#### 3.4.4. Robustness Test

**Figure 11:**
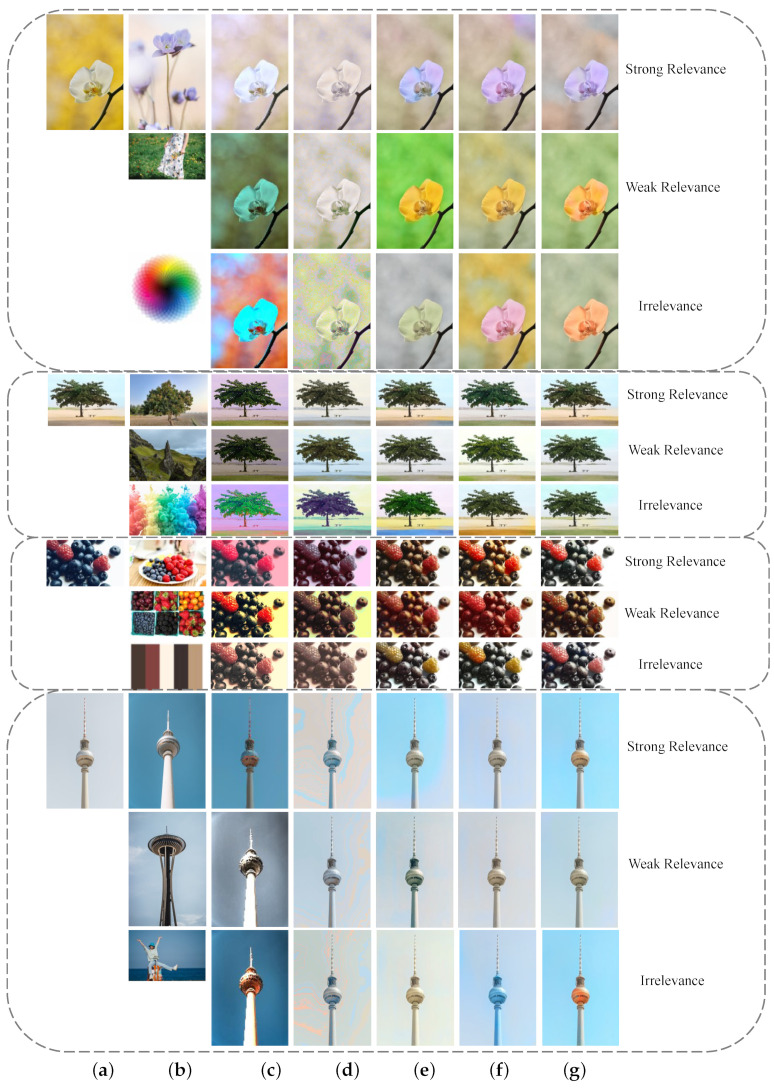
Qualitative results of an ablation study. There are a total of four groups of tests in the figure, and each group has three rows of images. The first, second, and third rows show color transfer results of image pairs of strong relevance, weak relevance, and irrelevance, respectively. The first column is the target image (TI), and the second column is the reference image(RI). (**a**) TI. (**b**) RI. (**c**) [1]. (**d**) [9]. (**e**) [18]. (**f**) [47]. (**g**) Ours.

One of the significant advantages of our method over traditional example-based colorization is the robustness of reference selection. It provides reasonable color regardless of whether the reference image is related to the target image or not. Figure 11 below perfectly illustrates the color transfer advantage of our method in different scenarios. The various relevances between the source image and the reference image are first classified into three cases. The first is strong relevance, that is, two images have high similarity in both the content and location of semantic objects. The second is weak relevance, which refers to high content similarity, but low relevance of object spatial configuration. The last is irrelevance, which refers to color palettes and image pairs with different content. A closer look at these four groups of images reveals that when the reference image is more similar to the target in its semantic content, the results are naturally more faithful to the reference image. In other cases, the result will degenerate to conservative coloring. This is caused by the perception branch, which predicts the dominant colors from the obtained on large-scale dataset.

To further visually demonstrate the advantages of our model, we perform a 1–5 ranking on the four sets of images in Figure 11. Among the five methods, the best results ranked first, followed by the second, third, fourth, and fifth. The ranking standard is mainly based on personal subjective evaluation, generally referring to several indicators such as color reflection, content preservation, naturalness, emotional expression, and fidelity. By analyzing the data in Table 7, it can be concluded that Xiao [18] ranks first with the most times, and our model ranks second closely, but our model ranks the top three times with 194 times, far higher than Lee [47] who ranks the second place with 168 times. In addition, Welsh [9] ranks last with the most times. By observing the coloring results in Figure 11, it can be seen that the color of the image is single and the fidelity is poor. Our model ranks last very few times, only 21, which is significantly better than most algorithms. At the same time, the number of times our model ranked in the last two is significantly less than that of other algorithms, which indicates that our algorithm has a good robustness and a low likelihood of generating poor results.

#### 3.4.5. Comparison of Running Time

Table 8 lists the average running time of these eight models tested on each image on the CPU/GPU, respectively. We found that the speed of processing an image on the GPU is faster than that on the CPU, especially the neural network model, which is at least 2 times faster than before. Whether running on the CPU or GPU, Iizuka [4] and Larsson [5] achieve image coloring faster than other algorithms, Xiao [18] is third, our model is fourth, Lee [47] and Zhang [6] ranked fifth and sixth respectively, and the two traditional algorithms, Reinhard [1] and Welsh [9], take the longest time. However, if the performance of the algorithm is compared according to the running time on the GPU, except Welsh [9], the other seven models can complete the task of image coloring within 5 s, and their performance is quite good. Therefore, the overall performance of our model is also excellent.

## 4. Conclusions

As an image enhancement processing method, image coloring aims to improve the accuracy of image coloring, visual effects, and precise application in image analysis. With the help of deep learning technology, this paper designs a color transfer method based on reference images. It has three main advantages: (1) The robustness of reference selection. Even if the two images or local regions are unrelated, this model can achieve a good result. (2) Flexible operation. Unlike previous deep learning frameworks, we can still manually control the results of coloring. At the same time, we can also color images and videos with automatic coloring. (3) The model has good transferability. It can be better applied to the color restoration of faded photos, medical image coloring, art restoration, remote sensing image enhancement, infrared image enhancement, and other fields. Our model also has three limitations: (1) Due to the perceptual loss function, we cannot generate colors that contain particularly strange or artist-formed colors. (2) The perceptual loss based on the classification network cannot penalize wrong colors in regions with less semantic importance, such as similar sand and grass textures, that is, the model cannot distinguish fewer semantic regions with similar local textures. (3) When there is a significant difference in brightness between the images, the color of the resulting image is not very faithful to the reference image. To mitigate this issue, our model enforces brightness consistency before performing color transfers.

## Figures and Tables

**Table 1:** Hardware and software information.

CPU	Memory	GPU	Operating System	CUDA Version
Intel E5-2620	62.8 G	Nvidia GTX 1080	Ubuntu 16.04 64 bit	9.0

**Table 2:** Specific classification of different datasets. ArtPhoto and FlickrEmotion have eight categories, namely: amusement, anger, awe, contentment, disgust, excitement, fear, and sadness. The number of images in each category of each dataset is different.

Dataset	Amusement	Anger	Awe	Contentment	Disgust	Excitement	Fear	Sadness	Summation
ArtPhoto	101	77	102	70	70	105	115	166	806
FlickrEmotion	320	322	356	305	329	389	397	357	2775

**Table 3:** **PSNR.** The data in Table 3, Table 4 and Table 5 are all from Figure 6, and the order of images is the same.

Methods	img_1	img_2	img_3	img_4	img_5	img_6	img_7	img_8	Average
[4]	51.603	37.856	43.141	36.346	50.68	50.393	48.551	36.606	44.397
[5]	32.334	34.404	28.825	31.526	31.901	37.084	37.155	28.781	32.751
[6]	36.153	46.403	44.948	41.76	40.8	44.002	42.623	39.729	42.052
[1]	19.784	13.567	17.724	11.208	18.745	22.553	15.228	20.907	17.465
[9]	26.303	33.814	34.987	33.099	30.394	33.54	38.892	22.768	31.725
[18]	33.511	38.521	37.372	41.723	21.119	39.658	39.865	36.09	35.982
[47]	23.296	38.505	36.594	42.657	36.823	32.711	26.123	36.415	34.141
Ours	34.411	40.494	39.598	42.728	33.108	38.384	47.099	36.993	39.102

**Table 4:** **SSIM**.

Methods	img_1	img_2	img_3	img_4	img_5	img_6	img_7	img_8	Average
[4]	0.891	0.954	0.948	0.943	0.948	0.954	0.96	0.857	0.932
[5]	0.887	0.954	0.948	0.935	0.948	0.955	0.959	0.851	0.930
[6]	0.892	0.953	0.947	0.943	0.948	0.954	0.959	0.858	0.932
[1]	0.496	0.704	0.786	0.72	0.89	0.709	0.867	0.817	0.749
[9]	0.881	0.951	0.948	0.937	0.944	0.954	0.959	0.846	0.928
[18]	0.889	0.954	0.948	0.943	0.882	0.955	0.959	0.857	0.923
[47]	0.683	0.954	0.948	0.943	0.948	0.953	0.934	0.857	0.903
Ours	0.89	0.954	0.948	0.944	0.947	0.958	0.966	0.857	0.933

**Table 5:** **QSSIM**.

Methods	img_1	img_2	img_3	img_4	img_5	img_6	img_7	img_8	Average
[4]	0.904	0.983	0.954	0.977	0.97	0.977	0.986	0.959	0.964
[5]	0.962	0.983	0.983	0.974	0.98	0.98	0.993	0.958	0.977
[6]	0.937	0.982	0.979	0.972	0.966	0.973	0.981	0.96	0.969
[1]	0.909	0.747	0.871	0.53	0.687	0.931	0.674	0.777	0.766
[9]	0.503	0.864	0.761	0.899	0.668	0.886	0.96	0.779	0.79
[18]	0.93	0.979	0.985	0.973	0.952	0.976	0.987	0.97	0.969
[47]	0.977	0.979	0.974	0.974	0.968	0.96	0.979	0.974	0.973
Ours	0.932	0.984	0.985	0.979	0.954	0.963	0.981	0.972	0.969

**Table 6:** **Colorful.** Generally, the subjective color evaluation of the image is between moderately colorful and quite colorful, that is, when the color degree *C* is between 33 and 45, the human eye will look more natural [45]. Among all the subjective color evaluations, the color difference of 12 between averagely colorful and moderately colorful is the smallest, so it can be considered that when the color similarity CS(res,ref) between the reference image and the result image is less than 12, the two images have similar colors. CS(res,ref) just said this color similarity between two images, and CS(res,ref)=∥C(res)−C(ref)∥, C(res), and C(ref) represent the color richness values of the resulting image and the reference image in turn.

Images	C(ref)	Three-Color	Five-Color	Seven-Color	Multi-Color	CS(res,ref)
IMAGE_1	84.232	49.815	77.926	83.764	80.1164	0.468
IMAGE_2	30.725	27.257	14.015	33.222	33.737	2.497
IMAGE_3	21.238	19.494	19.504	27.69	25.161	6.452
IMAGE_4	19.769	9.351	12.498	14.791	14.791	4.978

**Table 7:** Ranking results of user study. Fields 1, 2, 3, 4, 5 in the table represent the statistics of the number of times that each algorithm is ranked 1st, 2nd, 3rd, 4th, and 5th, respectively.

Methods	1st	2nd	3rd	4th	5th	Top 1	Top 3	Last 1	Last 2
[1]	51	48	51	38	52	51	150	52	90
[9]	26	12	25	75	102	26	63	102	177
[18]	72	42	31	48	47	72	145	47	95
[47]	30	81	57	54	18	30	168	18	72
Ours	61	57	76	25	21	61	194	21	46

**Table 8:** Comparison of running times. The data in Table 8 include the average running time of each image on the CPU/GPU, which is obtained by dividing the total time spent on testing the eight models on the ImageNet by the total number of images.

Methods	Type	Num.lter.	CPU Time (s)	GPU Time (s)	Speedup
[4]	CNN	1	4.576	1.015	4.508
[5]	CNN	1	4.262	1.036	4.114
[6]	CNN	1	6.173	2.452	2.518
[1]	-	1	7.36	4.25	1.732
[9]	-	1	23.58	12.13	1.944
[18]	CNN	1	5.79	1.34	4.321
[47]	DNN	1	7.34	1.85	3.97
Ours	CNN	1	6.87	1.77	3.881

## Data Availability

Not applicable.

## Abbreviations

The following abbreviations are used in this manuscript:

CNN	Convolutional Neural Network
RICT	Reference Image-based Color Transfer
PECE	Palette-based Emotional Color Enhancement
MRF	Markov Random Field
DNN	Deep Neural Networks
CEM	Color Estimation Model
PCNN	Progressive Convolutional Neural Network
LBP	Local binary pattern
HOG	Histogram of Oriented Gridients
PCA	Principal Component Analysis
BP	Back Propagation
PSNR	Peak Signal-to-Noise Ratio
SSIM	Structure Similarity
QSSIM	Quaternion Structural Similarity
CS	Colorfulness Similarity
